# CAR-NK Cells: From Natural Basis to Design for Kill

**DOI:** 10.3389/fimmu.2021.707542

**Published:** 2021-12-14

**Authors:** Muhammad Babar Khawar, Haibo Sun

**Affiliations:** ^1^ Institute of Translational Medicine, Medical College, Yangzhou University, Yangzhou, China; ^2^ Jiangsu Key Laboratory of Experimental & Translational Non-coding RNA Research Yangzhou, Yangzhou, China; ^3^ Molecular Medicine and Cancer Therapeutics Lab, Department of Zoology, Faculty of Sciences, University of Central Punjab, Lahore, Pakistan; ^4^ Laboratory of Molecular Biology & Genomics, Department of Zoology, Faculty of Sciences, University of Central Punjab, Lahore, Pakistan

**Keywords:** CAR-NK, CAR-T, clinical trials, HLA, KIR, cancer immunotherapy, receptors, tumor

## Abstract

Chimeric antigen receptors (CARs) are fusion proteins with an extracellular antigen recognition domain and numerous intracellular signaling domains that have been genetically modified. CAR-engineered T lymphocyte-based therapies have shown great success against blood cancers; however, potential fatal toxicity, such as in cytokine release syndrome, and high costs are some shortcomings that limit the clinical application of CAR-engineered T lymphocytes and remain to overcome. Natural killer (NK) cells are the focal point of current immunological research owing to their receptors that prove to be promising immunotherapeutic candidates for treating cancer. However, to date, manipulation of NK cells to treat malignancies has been moderately successful. Recent progress in the biology of NK cell receptors has greatly transformed our understanding of how NK cells recognize and kill tumor and infected cells. CAR-NK cells may serve as an alternative candidate for retargeting cancer because of their unique recognition mechanisms, powerful cytotoxic effects especially on cancer cells in both CAR-dependent and CAR-independent manners and clinical safety. Moreover, NK cells can serve as an ‘off-the-shelf product’ because NK cells from allogeneic sources can also be used in immunotherapies owing to their reduced risk of alloreactivity. Although ongoing fundamental research is in the beginning stages, this review provides an overview of recent developments implemented to design CAR constructs to stimulate NK activation and manipulate NK receptors for improving the efficiency of immunotherapy against cancer, summarizes the preclinical and clinical advances of CAR-NK cells against both hematological malignancies and solid tumors and confronts current challenges and obstacles of their applications. In addition, this review provides insights into prospective novel approaches that further enhance the efficiency of CAR-NK therapies and highlights potential questions that require to be addressed in the future.

## Introduction

**Figure 1:**
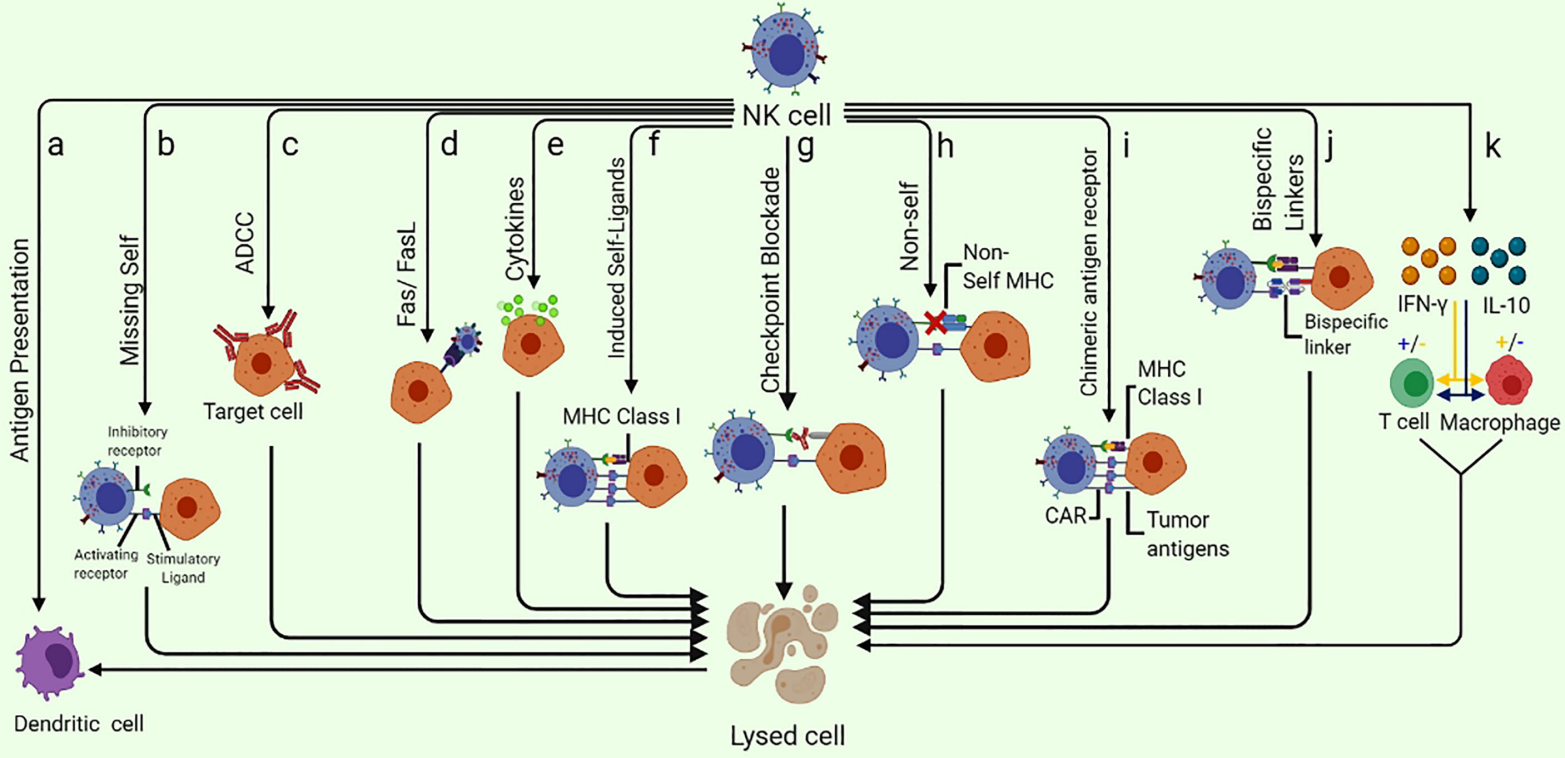
Different ways of NK-cell mediated tumor killing and immune system modulation: **(A)** NK cells are capable of enhancing the antigen presentation to T cells by killing the immature DC while promoting the IFN-γ and TNF-α mediated maturation of DC. **(B)** NK cells can specifically recognize the cells that lack the expression of self-MHC class I molecules (Missing-self). **(C)** ADCC can kill the target cell. **(D)** Fas/FasL pathway is a very effective NK cell-mediated cell killing as the binding of FasL to Fas results in delivering a “death signal” to the target cell that undergoes apoptosis shortly. **(E)** Cytokine pathway can exert anti-tumor potential as Cytokines such as NK cells secret several cytokines such as TNF-α. **(F)** NK cell receptors NKG2D are capable of recognizing the “induced-self” ligands that are express at a very high rate in response to the activation of tumor-associated pathways. **(G)** Checkpoint blockade may inhibit NK cell suppression by preventing the interaction of NK cell inhibitory receptors with their ligands. **(H)** As a result of adoptive NK cells transfer, the mismatch between donor and recipient, inhibitory KIRs, NK cells eliminate the allogeneic tumor cells that lack self-MHC. **(I)** CAR-NK cells designed specifically to target overexpressed tumor antigens are also useful in eliminating the specific tumor cells. **(J)** Specifically designed bispecific molecules are also being utilized to specifically eliminate tumor cells as these special molecules bind to activating NK cell receptors on one arm and tumor antigens on the other. **(K)** NK cells can enhance or diminish macrophage and T cell activities *via* IFN-γ and IL-10 production.

Natural killer (NK) cells are typical peripheral blood (PB) lymphocytes (5–10%) that were first identified in mice approximately 45 years ago (1). NK cell distribution varies among healthy tissues owing to the presence of unique chemokine receptors. NK cells are mainly found in the bone marrow, liver, spleen and PB; however, a few of them are also found in the lymph nodes (2). NK cells were initially described to exert cytolytic activity and directly kill tumor or virus-infected cells without any specific immunization, hence the name. Subsequently, NK cells were found to produce a large number of cytokines, especially interferon-gamma (IFN-γ), in several physiological and pathological conditions. They secrete various pro-inflammatory and immunosuppressive cytokines including tumor necrosis factor-alpha (TNF-α); interleukin-10 (IL-10) and growth factors such as granulocyte–macrophage colony-stimulating factor (GM-CSF), granulocyte colony-stimulating factor (G-CSF) and IL-3. Similarly, several chemokines, such as CCL2 (MCP-1), CCL3 (MIP1-α), CCL4 (MIP1-β), CCL5 (RANTES), XCL1 (lymphotactin) and CXCL8 (IL-8), are also produced by NK cells (3). The physiological role of the growth factors produced by NK cells remains unclear. The colocalization of NK cells in the inflamed area with other hematopoietic cells such as dendritic cells (DCs) is caused by their production of chemokines (4). Moreover, T cell responses in the lymph nodes are governed by NK cells. Stimulated NK cells release granzymes and perforin to mediate the killing of targeted cells (**Figure 1**) (5). Perforin increases permeability by creating a transmembrane (perforation) channel on the target cell, allowing granzymes to enter more easily and causing osmotic lysis. Granzymes at the cleavage site help in the lysis and killing of target cells (**Figure 1**). NK cells affect DCs indirectly, probably by secreting IFN-γ. The interaction of naïve T cells and NK cells entering the secondary lymphoid compartments from the site of inflammation plays a significant role in mediating T cell responses (6). As the first line of defense, NK cells prevent pathogen invasion and tumorigenesis. After viral infection, NK cells are activated quickly, without prior sensitization, to avoid anomalous cells and infection (7). Compared with autologous NK cells, allogeneic NK cells are usually more potent and highly toxic against tumors (8). NK cells are categorized into subpopulations based on their functional attributes and maturation levels. Advancements in the profound understanding of tumor immunology have made NK cell biology, especially its clinical applications, an interesting focus area for research in recent years. A few features that make NK cells unique for their use in future immunotherapies are described below.

### Receptors and Their Mechanisms for Regulation of NK Cells

**Figure 2:**
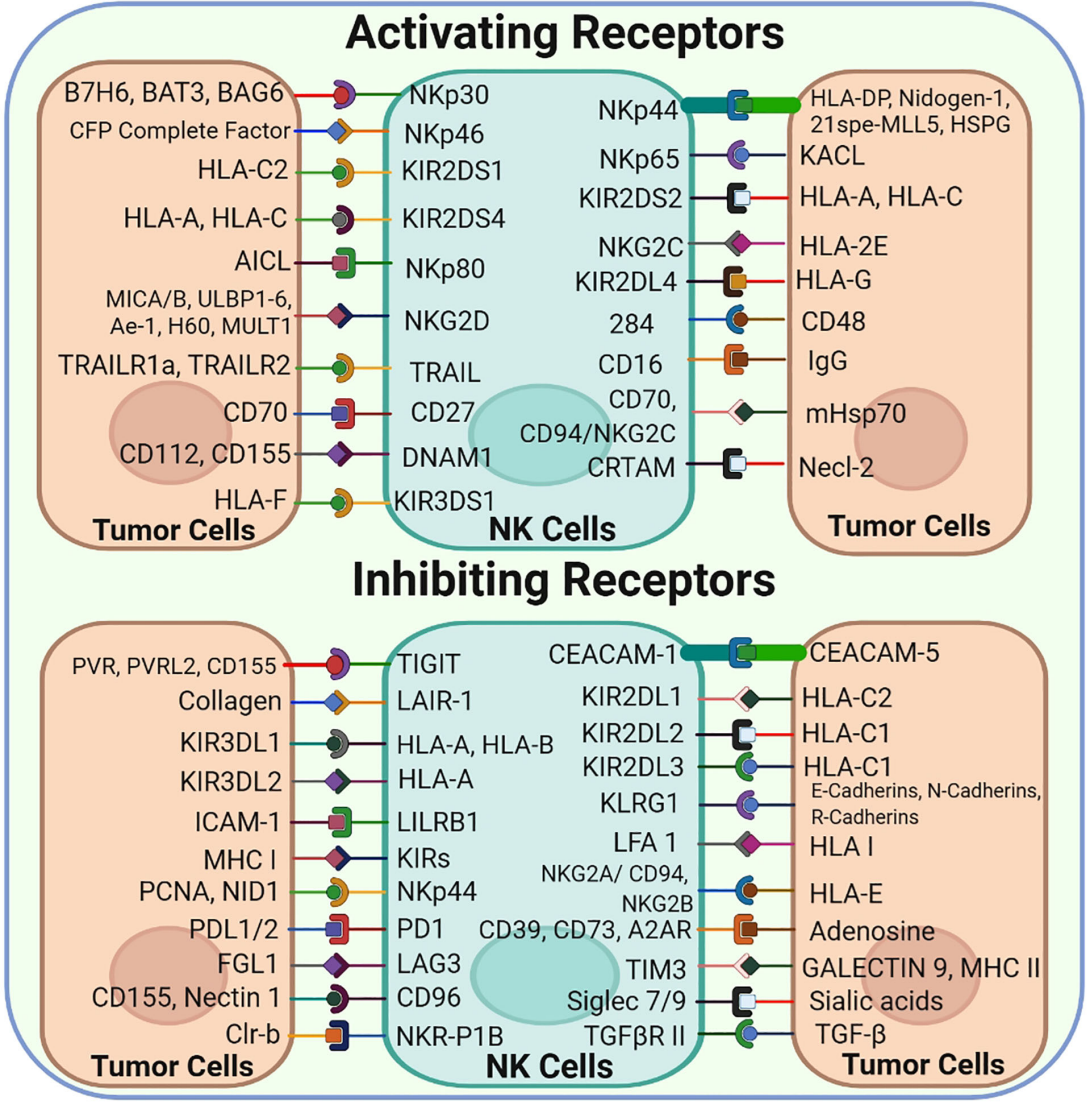
NK-Cell Surface Receptors and their corresponding ligands: NK cells express a vast array of both Activating and inhibitory cell surface receptors that interact with their corresponding ligands found on the tumor cell surface.

Several cytoplasmic membrane receptors, including activating, inhibitory, cytokine and chemokine receptors, are expressed on NK cells (**Figure 2**) (5). The classical and non-classical major histocompatibility complex (MHC) class I molecules present on normal cells are recognized by inhibitory or activating receptors expressed on NK cells. A balance between the signaling of inhibitory and activating receptors regulates the activation and role of NK cells (9).

MHC molecules or other inhibitory receptors that recognize the corresponding ligands are downregulated in tumor cells; however, NK cells are activated and subsequently kill tumor cells *via* the so-called ‘missing-self’ mechanism (**Figure 1**) (10). Normally, MHC molecules present on the surface of healthy cells act as ligands for inhibitory receptors and help in establishing the self-tolerance of NK cells. However, cells may lose these molecules owing to tumor development, resulting in reduced inhibitory signals to NK cells. Another major mechanism based on receptor–ligand interaction that triggers NK cell activation is called ‘induced-self’ mechanism. Several activating receptors, including NKG2D and activating killer immunoglobulin-like receptors (KIRs), are capable of recognizing their corresponding interactive ‘induced-self’ ligands that either lack or express merely on healthy cells but highly express on cancer cells in response to the activation of tumor-associated pathways (11, 12). Consequently, these ‘missing-self’ and ‘induced-self’ changes are characterized by significant cellular stress in the form of cellular ageing, DNA damage response and tumor suppressor genes that stimulate the robust expression of ligands for activating receptors. NK cells are activated under the influence of these activating receptors and eliminate target cells either directly *via* NK cell-mediated cytotoxicity or indirectly *via* pro-inflammatory cytokine-mediated killing (13).

Antibody-dependent cell-mediated cytotoxicity (ADCC) is another way to target tumor cells. NK cells are characterized by the abundance of CD16 (FcγRIIIA) that serves as a receptor for IgG1 and IgG3 and is indispensable for NK cell-mediated ADCC. As a prototype NK cell-activating receptor, CD16 can trigger the cytotoxicity and secretion of cytokines and chemokines, thereby imparting antitumor activity to NK cells (14–16). A few circulating monocytes and macrophages also express CD16 (17), which is comprised of two extracellular Ig domains, a cytoplasmic tail and a transmembrane domain. The transmembrane domain helps CD16 to bind with the CD3ζ and FcϵRIγ chains, resulting in the formation of immunoreceptor tyrosine-based activation motif (ITAM)-containing subunits that associate these subunits with the intracellular signal transduction pathways to regulate the activation of numerous transcription factors and reorganization of cytoskeletal elements (13, 15). In NK cells, such pathways mediate ADCC characterized by target-oriented secretion of cytotoxic granules (perforin and granzymes) and Fas ligands and the involvement of TNF-related apoptosis-inducing ligand (TRAIL) death receptors (18, 19). Furthermore, CD16 engagement supports the survival and proliferation of NK cells (20, 21) and stimulates cytokine and chemokine secretion, leading to the recruitment and activation of tumor-infiltrating immune cells (22, 23).

A major pathway involved in NK cell-mediated cytotoxicity is known as the Fas/FasL pathway (24). Fas (Apo-1 or CD95) and Fas ligands (FasL or CD95L) are type-I and type-II transmembrane proteins, respectively, and belong to the TNF family. When FasL binds to Fas, it sends a ‘death signal’ to the target cell, causing it to undergo apoptosis. The secretion of several cytokines (e.g. TNF-α) from NK cells is referred to as the cytokine pathway (5), which kills target/tumor cells.

The modulation of macrophages and T cell responses can also kill target cells. NK cells can enhance or diminish macrophage and T cell activities *via* IFN-γ and IL-10 production (25). These cytokines lead to the release of hydrolases from the lysosomes of target cells by altering the phospholipid metabolism of the cell membrane. This altered metabolism activates endonucleases that degrade the genomic DNA of target cells (26). NK cells mediate immune responses *via* cross-talk between NK cells and DCs. NK cells can enhance antigen presentation to T cells by killing immature DCs and promoting maturation of DCs mediated by IFN-γ and TNF-α (25). Similarly, another potential mechanism to kill target cells is checkpoint blockade that manifests by preventing the interaction of inhibitory receptors with their respective ligands. Consequently, a mismatch between the KIRs and MHC class I molecules of donor cells and those of recipient NK cells may initiate the elimination of target cells. In addition, genetically expressed activating CARs on NK cells can specifically bind to tumor antigens. Interestingly, bispecific molecules are also being exploited to bind with the activating receptors on NK cells and tumor antigens simultaneously to stimulate NK cell-mediated tumor lysis. Usually, various signals against inhibitory receptors, instead of activating receptors, maintain homeostasis in the host (27). Inhibitory receptors act as checkpoints, such as those in T cells, to control NK cell activation (28). Overexpression of PD-1, TIM-3, inhibitory KIRs, NKG2A and T cell immunoreceptors with Ig and immunoreceptor tyrosine-based inhibition motif domains (TIGIT) on NK cell surface has been reported in the tumor microenvironment (TME) and viral infections (29). The interaction of inhibitory receptors with the respective ligands on DCs, regulatory T cells (Tregs), tumor cells and infected cells generates signals that regulate the activation, effector function and even the subsequent functional exhaustion of NK cells (30). NK cells can, therefore, be activated *via* checkpoint receptor inhibition. For instance, anti-TIGIT and anti-NKG2A mAbs have shown a superior anticancer activity because they can restore the antitumor activities of both NK and T cells. Recently, a combination of cetuximab (anti-EGFR) and monalizumab (anti-NKG2A) demonstrated an objective response rate of 31% against head and neck squamous cell carcinoma (31).

Identification of HLA class I-specific inhibitory receptors, especially KIRs, reveals the mechanisms underlying the killing of tumor cells by NK cells. The next section provides a quick overview of KIRs.

### Killer Immunoglobulin-Like Receptors

KIRs specifically expressed on human NK cells, which are encoded by LRC and found on human chromosome 19. They can bind to MHC class I molecules present on target cells. Both inhibitory and activating receptors are found in the KIR family. Inhibitory KIRs use the immunoreceptor tyrosine-based inhibition motifs (ITIMs) located in the extended cytoplasmic tails to transmit inhibitory signals. However, activating KIRs possess short tails and use DAP12 or FcγR as an adaptor molecule to transduce activating signals to NK cells (32). There are two (KIR2DL) or three (KIR3DL) extracellular Ig domains in inhibitory KIRs that equip them with their functional specificity for HLA molecules. Therefore, KIRs are highly sensitive to any change in HLA molecules during cancer and viral infections, as recently reviewed in detail (33). Normally, KIRs are expressed constitutively; however, inhibitory signals naturally predominate and suppress the activating signals and fine-tune NK cells to protect healthy cells, also known as NK cell self-tolerance. The phenomenon of self-tolerance was recognized to be regulated by the binding of some powerful inhibitory receptors (such as KIRs and NKG2A) to MHC class I molecules. Reduced expression of MHC class I molecules in malignancies (missing self) was presumed to prevent this inhibition and permit self-reactivity (34). KIR–HLA mismatch is also a significant factor that should be considered for cancer immunotherapy. Each KIR recognizes a particular HLA allotype as an inhibitory ligand; KIR2DL1, KIR2DL2/3 and KIR3DL1 specifically bind to group 2 HLA-C, group 1 HLA-C and HLA-Bw4, respectively. Therefore, a superior and more potent antitumor activity can be exhibited by recipients lacking specific HLA allotypes for inhibiting NK cells. Several previous studies have reported that KIR–HLA mismatch between a donor and a recipient results in a higher antitumor potential (35–37). Furthermore, to attain the best antitumor effects, the actual expression of KIRs should be considered because they are usually expressed in stochastic combinations (38). Interestingly, single-KIR^+^ allogeneic NK cells that do not encounter any HLA-inhibitory signal mediate the antitumor activity (39). NK cells recover first from allogeneic hematopoietic stem cell transplantation (HSCT); however, it takes approximately 3 months for the NK receptor repertoires to reconstitute (40, 41), and single-KIR^+^ NK cells are not completely functional until then (39). However, KIR-mismatched allogeneic NK cells might be rejected due to MHC mismatch. For instance, acute kidney transplant rejection resulting from KIR/HLA polymorphism was found in a KIR/HLA genotype study (42). Similarly, in a phase II clinical trial (NCT00703820), KIR–HLA-mismatched allogeneic NK cells were found ineffective against medium-high-risk acute myeloid leukemia (AML), possibly owing to their insufficient number and poor persistence (43).

NK cells are educated and licensed by inhibitory receptors that recognize MHC molecules to respond to MHC-I-deficient cells. The inhibitory receptors are expressed during NK cell development and ensure their functional competence. NK cells that lack these inhibitory receptors cannot undergo education and licensing and remain hyporesponsive. For instance, KIR3DL^+^ NK cells of an individual having HLA‐Bw6 are more hyporesponsive than those of an individual having HLA‐Bw4 because Bw4 serves as a ligand for KIR3DL1. Therefore, the strength of inhibitory signals may influence NK cells (44, 45). Moreover, only a few NK cells that lack self-reactive receptors are anergic because NK cell ‘licensing’ or ‘education’ during maturation imparts this property (46). Inhibitory KIRs are downregulated whereas activating KIRs are upregulated in several cancer types, such as skin cancer, lymphoma, leukemia, breast cancer and biliary cancer (47). Changes in the expression patterns of these KIRs may aid tumor escape by inhibiting NK cell activation and subsequent anticancer activity.

Inhibitory KIRs were the first to be considered the checkpoint receptors in NK cells (48). Because mAbs can recognize inhibitory KIRs, they enhance the anticancer activity of NK cells by blocking their signal transduction. Therefore, mAbs are potential therapeutic candidates that are safer and have fewer side effects as compared with other therapeutic approaches, which have been employed in several clinical trials against several tumor types (49).

The outcomes of earlier studies employing autologous NK cells were disappointing owing to self-HLA molecules mediated repression (50–52). With the establishment of KIR–ligand mismatch in transplantation, there came a boom in the application of allogeneic NK cells in both HSCT and non-HSCT (53–55). Compared with autologous NK cells, allogeneic NK cells does not face a repression from self-MHC molecules. Furthermore, multiple studies have indicated that infusing haploidentical NK cells into patients with AML to investigate the missing-self mechanism is safe and can induce substantial clinical activity (53–55). Adoptive transfer of haploidentical NK cells to patients with AML was found to be safe because it did not result in graft-versus-host disease (GVHD), and IL-2 administration increased their persistence by approximately 4 weeks (54).

### NK Cell Sources and Expansion

NK cells are commonly used in cell-based immunotherapies for hematological malignancies (56); however, they have also shown some hope for the treatment of solid tumors in preclinical studies (discussed in detail in later sections of the manuscript). Previously, IL-2-stimulated autologous NK cells were used in NK cell immunotherapies; however, the binding of KIRs on NK cells to the ligands of endogenous HLAs present on cancer cells leads to suppression, limiting the clinical outcomes. This suppression can be prevented by transferring allogeneic or haploidentical NK cells having KIR–ligand mismatch owing to missing-self recognition of tumor cells (35). Consequently, allogeneic NK cell mixtures are considered safer and may help to eradicate leukemia regression and prevent GVHD, especially when HLA class I molecules are downregulated by cancer cells. Therefore, using allogeneic NK cells as specific unified sources of therapeutics may facilitate large-scale GMP-based ‘drug’ production, leading to overwhelming treatment effects.

**Figure 3:**
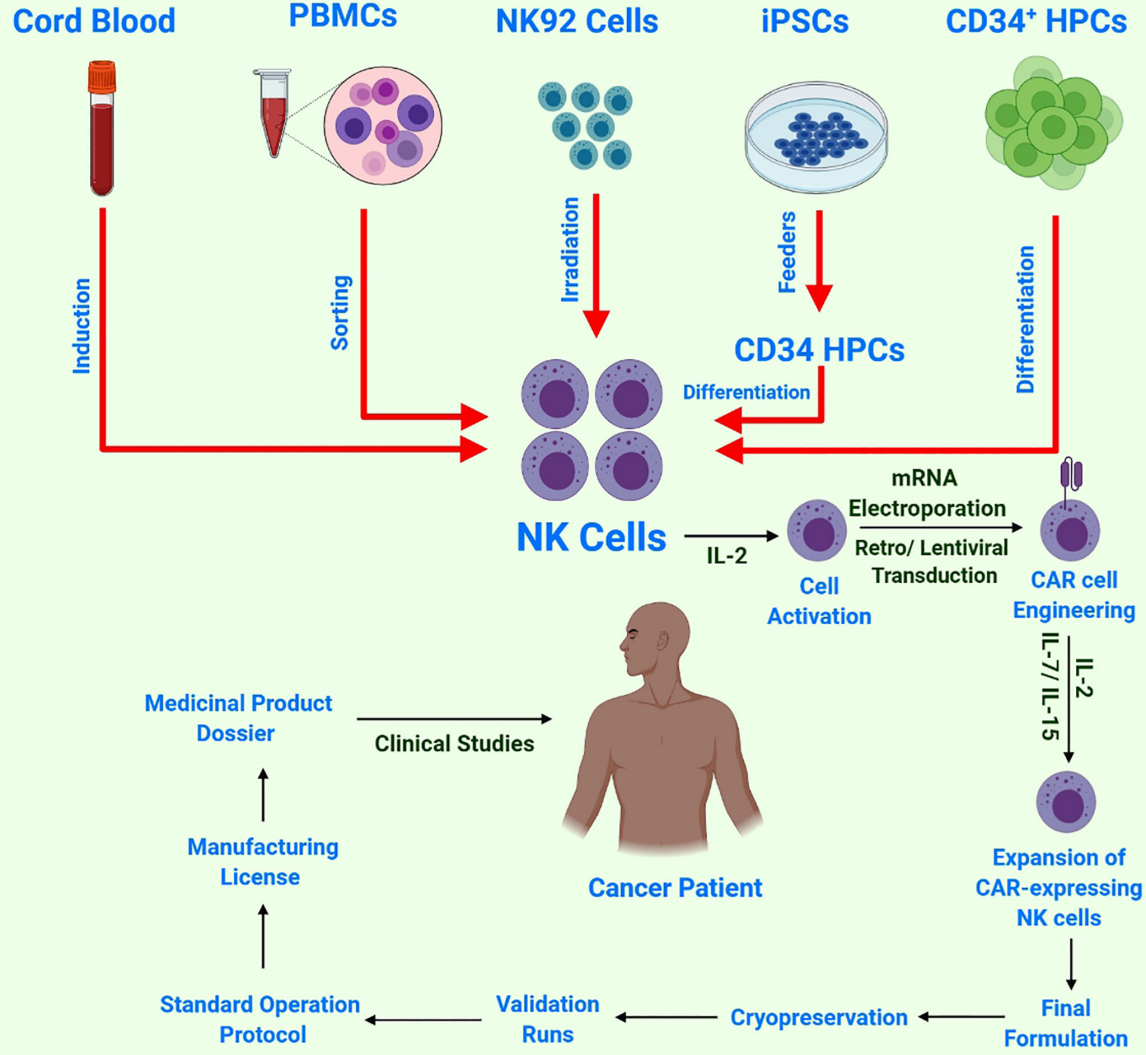
Schematic of NK cell sources and CAR-NK cell/immune therapy workflow: NK cells harvested from multiple sources, followed by NK-cell activation by IL-2 and subsequent transduction with a construct encoding CAR. Next, these reprogrammed CAR-NK cells are expanded *ex-vivo* and passed through strict quality control testing before cryopreservation and subsequent validation and approval for final clinical administration.

Various NK cells obtained from different sources are under investigation in clinical settings (**Figure 3**) (57). Induced pluripotent stem cells (iPSCs) can be transformed into all cell types, including immune cells. Several differentiation methods are now available for producing immunological effector cells from iPSCs. Moreover, a quick, powerful and well-defined procedure for the differentiation of T and NK cells has been recently developed (58). The hematopoietic stem cell (HSC) stage is one of the most important intermediate phases in the development of iPSCs to fully differentiated immune cells. Adult HSCs produce all types of blood cells. Adult bone marrow and the umbilical cord of neonates are the two common sources of HSCs. The currently available protocols are not completely optimized to minimize HSC fatigue and differentiation; therefore, it is necessary to develop protocols to increase HSC multiplication and pluripotency for improved immunotherapeutic applications. It is encouraging that researchers are working towards developing a more targeted and efficient HSC growth medium (59). Owing to their adaptability of growth conditions, iPSCs are an alternative or complementary strategy to achieve a large number of HSCs (60, 61).

Moreover, to overcome the critical problems encountered thus far, iPSCs have been used to create ‘off-the-shelf’ CAR-NK cells by taking advantage of iPSCs as a renewable source for NK cells, thus allowing for constant, precisely defined immunotherapy and genome editing in different anticancer modes of action. These CAR-iNK cells may be cryopreserved and supplied on demand to each patient, significantly decreasing the manufacturing costs. Moreover, NK cells from PB were found more advantageous in terms of safety and cytotoxic potential against tumors (62).

Cytokines have been reported to induce NK cells into a memory-like phenotype having a prolonged life expectancy under *in vivo* conditions (63). Memory-like NK cells undergo differentiation and result in an amplified IFN-γ yield and enhanced cytotoxicity against leukemia after brief preactivation with IL-12, IL-15 and IL-18, both *in vitro* and *in vivo*. The aforementioned memory-like NK cells have significantly depicted efficacy in AML, wherein 5 out of 9 patients showed complete remission (CR) (64). Briefly, 13 patients underwent several treatments that included memory-like NK cells at three distinct dosages, i.e. levels 1, 2 and 3. Of the 13 patients, 4 achieved CR/CR with incomplete blood count recovery (CRi) and 1 achieved morphologic leukemia-free state (MLFS), with an overall response rate of approximately 55% and a CR/CRi rate of 45%. Interestingly, IL-15-stimulated NK cells exhibited significant therapeutic efficacy against solid tumors. Of the 6 patients with refractory solid tumors, 4 exhibited a positive clinical response after receiving haploidentical stem cell transplants, 1 exhibited high partial remission, whereas 2 exhibited only partial remission (65). Unfortunately, the poor expansion of NK cells in patients with tumors makes their clinical application limited, and owing to strict evaluation of donors, those contributing allogeneic NK cells are quite difficult to find. Fortunately, the results of adoptive NK cell therapy have proved both autologous and allogeneic NK cells considerably safer and more tolerable (66).

NK cell expansion is stimulated by proliferative cytokines (IL-2 and IL-7/IL-15) (67). Similarly, the use of artificial antigen-presenting feeder cells (aAPC) in a culture system that facilitates gas permeability has been proposed as a new approach for growing NK cells from cryopreserved cord blood (CB) units. CB-NK cells expanded 1848-fold from fresh blood and 2389-fold from cryopreserved CB after 14 days, with >95% purity for NK cells (CD56^+^/CD3) and <1% for CD3^+^ cells (68). The co-culturing of NK cells using irradiated feeder cells in media supplemented with IL-2 and IL-15 is used to harvest a large number of NK cells, although an expansion system without the use of feeder cells is already available that used anti-CD3 antibodies (OKT-3) for NK cell expansion. RPMI8866, the Epstein–Barr lymphoblastoid cell line (EBV-LCL) and K562 are the most common feeder cell lines exploited for NK cell expansion (69). Activation by NK-sensitive K562 cells has been found to enhance NK cell proliferation with IL-2, IL-15 and IL-21 (67). K562 are good activators of NK cells owing to the lack of HLA expression and loss of inhibitory signals. Recently, significantly high NK cell expansion rates were observed with genetically engineered (GE) feeder cells. The co-expression of IL-15 and 4-1BBL *via* membrane-bound K562 was found to act collaboratively to strengthen the activation capability of K562-specific NK cells and resulted in remarkable expansion of PB CD56+CD3−NK cells devoid of any satellite T lymphocyte build-up (70). Ojo et al. (71) recently developed an NK cell feeder cell line named ‘NKF’ by overexpressing membrane-bound IL-21. This cell line could induce robust and sustained proliferation (>10,000-fold expansion at 5 weeks) of highly cytotoxic NK cells. Compared with IL-2-activated non-expanded NK cells, the expanded NK cells were highly cytotoxic against several types of malignancies. Expanded NK cells were also effective in mouse models of human sarcoma and T cell leukemia (71). Copik et al. reported a unique method for efficient expansion of NK cells with the help of plasma membrane (PM) vesicles. Briefly, they cultured K562-mb15-41BBL cells and optimized the formation of PM vesicles with a higher level of 41BBL using the nitrogen cavitation method. The optimized PM vesicles (PM 15) resulted in a 293-fold expansion of NK cells after 12–13 days as compared with 173-fold expansion achieved with live feeder cells. NK cells not only expanded better after stimulation with PM-mb15 41BBL vesicles but also exhibited phenotypes, surface receptors and superior cytotoxicity comparable with those of NK cells expanded with live feeder cells (72). Furthermore, PM21 particles were prepared to employ K562-mb21-41BBL cells. Briefly, PBMCs were cultured for 28 days with PM21 particles, resulting in >90% NK cell expansion by day 14 and an exponential expansion of 100,000-fold by day 28 (73). Similarly, a method for better expansion of memory NK cells was described recently. Briefly, NK cells are preactivated using stimulatory cytokines, and these preactivated NK cells were then expanded using a vesicle having NK cell effector agents such as PM21 particles, EX21 exosomes or FC21 feeder cells (74).

Remarkably, iPSC-NK cells were reported to produce a large number of homogenous NK cells; therefore, they can be banked and stored (75). aAPC-expanded PB-NK and iPSC-NK cells exhibited a higher antitumor potential *in vivo* when compared with PB-NK cells that had undergone overnight activation (76). NK cell lines, especially NK-92, have demonstrated better antitumor efficacy and offered more advantages as an ‘off-the-shelf’ approach because they have reduced toxicity and lack most inhibitory KIRs, except a very mild expression of KIR2DL4 (77), as compared with other NK cell sources and have successfully entered multiple clinical trials (78). Moreover, a high dose (10^10^ cells) was found to be safer for effective results in patients with melanoma and lung cancer (78).

## Chimeric Antigen Receptor

**Figure 4:**
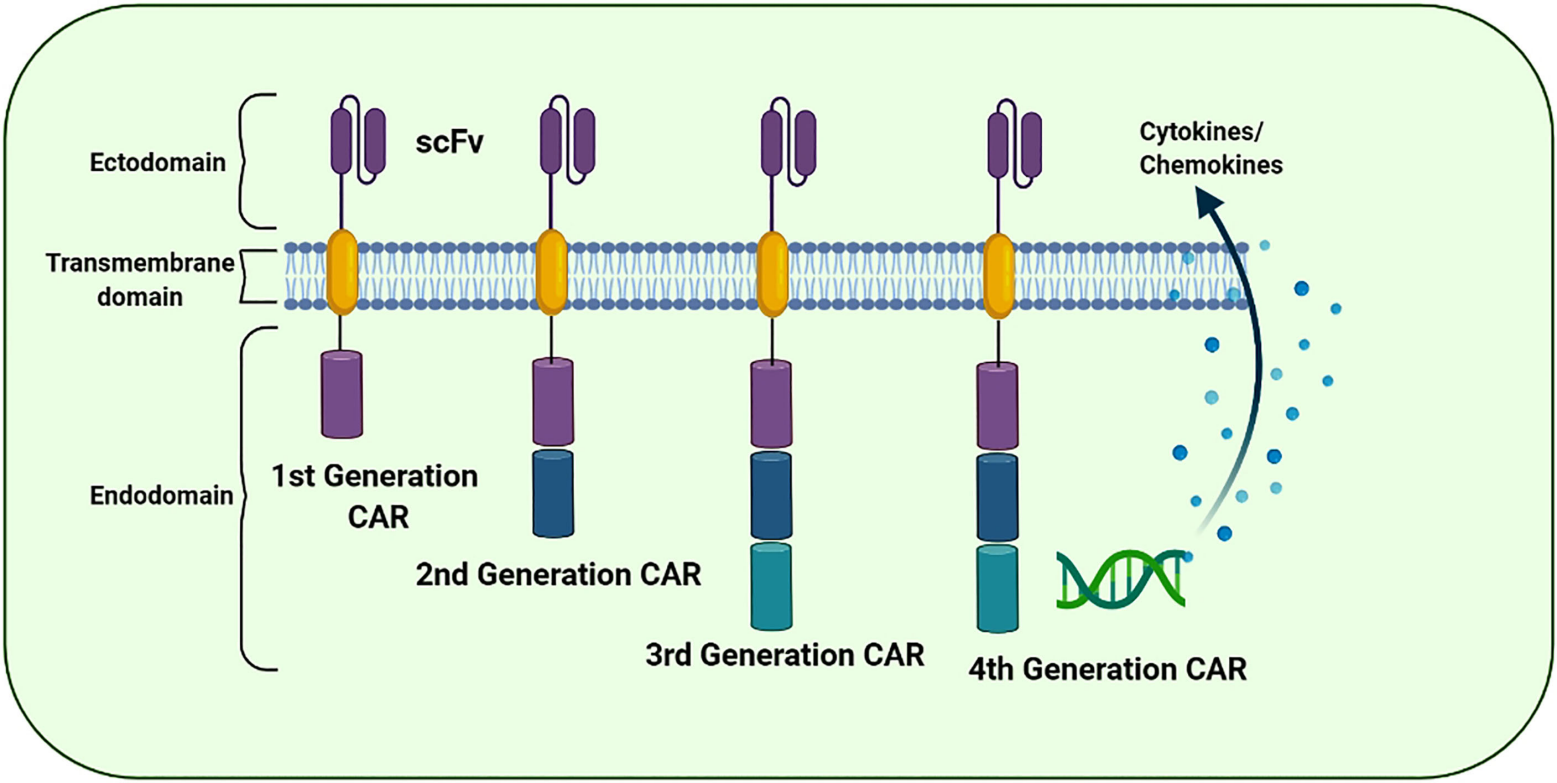
The evolution of the chimeric antigen receptor (CAR) structure over time: The structural components of 1^st^, 2^nd^, 3^rd^, and 4^th^ generation of CAR. The CAR “generations” denote the number and composition of the intracellular signaling domains. The 1^st^ generation’s CARs failed to deliver cell proliferation signals for the retention of anti-cancer potential. However, 2^nd^ and 3^rd^ generation CARs have CD28, CD134 (OX40), and CD137 (4-1BB) to promote the anti-tumor potential. The 4^th^ CAR generation is designed to secrete cytokines to further improvise the therapeutic activity of the CAR-based immunotherapies.

CAR is a fusion protein created intentionally and is found on T cell receptors (TCRs). It contains an extracellular antigen recognition domain and various intracellular signaling domains. Cross-reactivity of TCRs has only been observed in receptors with a supra-physiological affinity for cognate antigens. Moreover, CARs are capable of overcoming resistance observed in several malignancies because they, unlike TCRs, engage molecules independent of MHC recognition and antigen presentation by target cells. CAR constitutes an extracellular antibody-like region known as the single-chain variable fragment (scFv) which is intended to bind to a specific antigen and a hinge region of flexible lengths depending on the vicinity of the perceived epitope situated on the exterior of the target cell. It also contains a transmembrane domain along with one or more co-stimulatory domains and a signaling domain capable of persuading cytotoxicity as a consequence of antigen binding (79) (**Figure 4**). scFvs targeting a particular molecule can be derived from several sources such as murine or humanized antibodies. Furthermore, phage display libraries can be screened for the identification and synthesis of scFvs (80). The specificity of CARs is usually determined by the antibody scFv region; however, NK cell receptors have also been used for this purpose. The ligand-binding domains of scFv region are coupled with extracellular, transmembrane and signaling domains of other cell proteins using recombinant DNA technology. NK cells may identify distinct types of tumor cells through numerous cell surface receptors. The ligands recognized by NK cell receptors are present on various tumor cell types, making them promising targets for tumor-targeting therapies. The NK cell receptor NKG2D and its ligands have attracted significant attention for being involved in a possible tumor-killing approach. Various tumor cells express NKG2D ligands, exhibiting comparative selection between ligand expression on tumor and healthy cells (81). Previous studies have reported several tumor antigen-binding domains as CAR extracellular domains. In addition to the antigen-binding domain, a hinge region is located extracellularly along with the transmembrane domains of CD8 or IgG4. The intracellular signaling domain is the most important section of CARs because it decides the functionality of CAR. The most common intracellular domains are CD3, CD28, 4-1BB or OX40, which are premeditated to increase T cell activation. CARs that can directly identify CAR-targeted antigens are involved in T cell activation, proliferation, cytokine production and cytotoxicity against tumor cells that express CAR-specific antigens and are synthesized by genetically engineered T cells (82).

## CAR Generations and CAR-T Therapy

**Figure 5:**
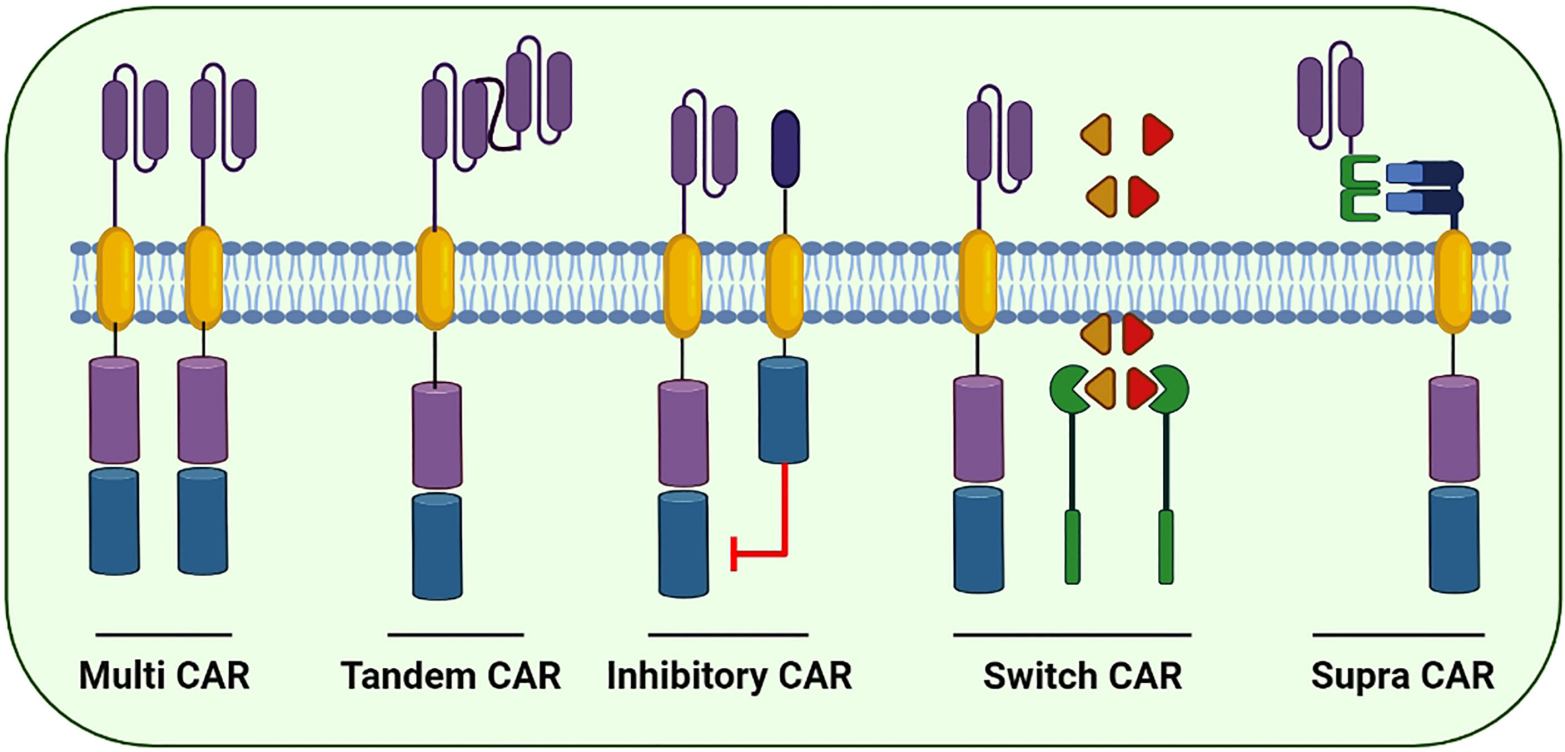
Few of the next generation CARs to better cope with the immune escape and improve the cytotoxic potential of CAR-based immunotherapies: Multi CARs are equipped with two or more separate CARs expressing various ScFvs to target the cancer cells. Tandem CARs are equipped with two different scFvs in a single CAR molecule. Upon antigen recognition in healthy cells, Inhibitory CARs tend to inhibit immune cell activation. In switch CARs, certain chemicals capable of dimerization with the iCasp9 are conditionally administered to activates the downstream caspase molecules leading to the apoptosis of CAR-expressing cells. Supra CARs are equipped with two split structures; the antigen-binding domain (zipFV) and function domain (zipCAR) that upon binding activates the CAR-expressing cells.

CAR ‘generations’ generally denote the number and composition of the intracellular signaling domains. Currently, the fourth generation of CARs is under technical development because its use in clinical practice has not yet been approved (83). The first-generation CARs contained a domain equipped with scFv to recognize tumor antigens and an activation motif (ITAM, generally CD3ζ) (84). Unfortunately, these CARs could not support persisting cell proliferation indicators for resuming the anticancer activity. Furthermore, the second- and third-generation CARs were equipped with CD134 (OX40), CD28 and CD137 (4-1BB) to enhance their propagation and cytotoxic potential (85). In the subsequent (fourth) generation, CARs are designed to secrete cytokines and are usually provided with more than one co-stimulatory molecule such as CD134, CD28 or CD137 to enhance antitumor potential by stimulating the innate immune system (86). In addition, several next-generation CARs have also been developed and are in an experimental phase (**Figure 5**).

The first-ever CAR-T cell therapy (Kymriah; Novartis) was authorized in 2017 by the United States Food and Drug Administration (FDA) to treat B-cell acute lymphoblastic leukemia (ALL) (87, 88). Subsequently, another CAR-T cell therapy (Yescarta; Kite Pharma) was developed and approved to treat non-Hodgkin’s lymphomas (89). Several hematological malignancies, including lymphoma, chronic lymphocytic leukemia (CLL) and ALL, are treated using CAR-modified T cell therapy, which has shown extraordinary results. Notably, feedback rates of 70–90% have been achieved in patients with ALL treated with CD19-targeting CAR-T cell therapy (90). Furthermore, the four CAR-T therapies approved by the FDA include Breyanzi, the first cell therapy product of Bristol Myers Squibb (BMS); Kymriah (tisagenlecleucel) by Novartis and Yescarta (axicabtagene ciloleucel) and Tecartus (brexucabtagene autoleucel) by Gilead/Kite. Tecartus, which was licensed in the United States and Europe in 2020, is currently only used to treat mantle cell lymphoma (MCL), which is not among approved indications of other CAR-T therapies. The FDA has authorized Abecma (idecabtagene vicleucel; ide-cel) for treating adult patients with relapsed or refractory multiple myeloma.

The prime focus of the formulation of fourth-generation CARs is to address the prevailing challenges and constraints in its clinical applications. Next-generation CAR constructs can be further divided into subgroups including tandem, combinatorial, ON-switch, inhibitory, universal and T cells reprogrammed as global cytokine killing (TRUCK) CARs based on their function. Small molecules are required for the assembly of ON-switch CARs that promote controlled CAR activation *via* drug administration (**Figure 5**) (91). Similarly, another fragmented CAR design is of universal CARs that can target numerous cancer types by exchanging antigen-specific regions with the same TM and an intracellular signaling construct (92). Furthermore, OR-gate CARs present a novel strategy to prevent tumor escape by attaching two scFv domains to distinct targets adhered either to a single TM along with an intracellular domain (tandem CAR) or a complete dual CAR construct build on a single cell (dual CAR) (93), and T cells can be activated by signals transduced by either scFv. Similarly, AND-gate CARs also involve two scFvs; however, they require both antigens to be present on the same cell before signal propagation. This approach is unique in targeting non-tumor-specific antigens because the binary execution of two antigens, namely, combinatorial CAR and synNotch receptor, helps in achieving tumor specificity (94). Furthermore, a novel CAR design, known as TRUCK-CAR, carries a transgenic ‘payload’ to target solid tumors. These CARs modulate TME because they can induce cytokine transgene products such as IL-12 and mediate the release of ‘payload’ at the tumor site (86). In addition, an extracellular inhibitory domain is fused with an activating intracellular CAR domain to convert an immunosuppressive signal to an activating signal in inhibitory CARs (**Figure 5**) (95). Moreover, to ensure the safety of CAR-T therapy, a suicide switch has been developed that activates in case of any adverse effects; however, this safety measure is found in only 10% of the current clinical trials (96).

The development of CAR-T technology has revolutionised cancer therapy. However, currently available CAR-T technology presents significant barriers to its widespread acceptance. Several issues include high cost, patient-oriented manufacturing, inconsistency in CAR-T production and function because the immune system of patients is intrinsically weakened and potential side effects. However, NK cell treatment has the potential to address some of these problems. Multiple anticancer receptors are used by NK cells that do not induce GVHD. However, their decreased *in vivo* lifespan requires numerous doses, enhancing the chances of rejection. Currently available CAR therapies are susceptible to checkpoint blockades and other immunosuppression strategies that reduce their ability to kill *in vivo*.

## NK Cells: Alternative Host Cells for CAR Therapy

The success of CAR-T therapy in clinical trials has led to the development of CAR-NK cells. Extracellular, transmembrane and intracellular signaling domains are present in CAR-NK cells as they are in CAR-T cells. CAR-NK cells often have CD3 as their initial signaling domain and CD28 or CD137 (4-1BB) as a costimulatory domain to form an intracellular signaling motif. NK cells increase their cytotoxic capability and cytokine production through two more costimulatory molecules, namely, NKG2D and CD244 (2B4) (97). Owing to more enhanced tumor-specific targeting and cytotoxicity than those of CAR-T cells, CAR-modified NK cells have been used to target cancer cells (98). Notably, NK cells can serve as another candidate along with T cells in CAR-targeted immunotherapies.

**Figure 6:**
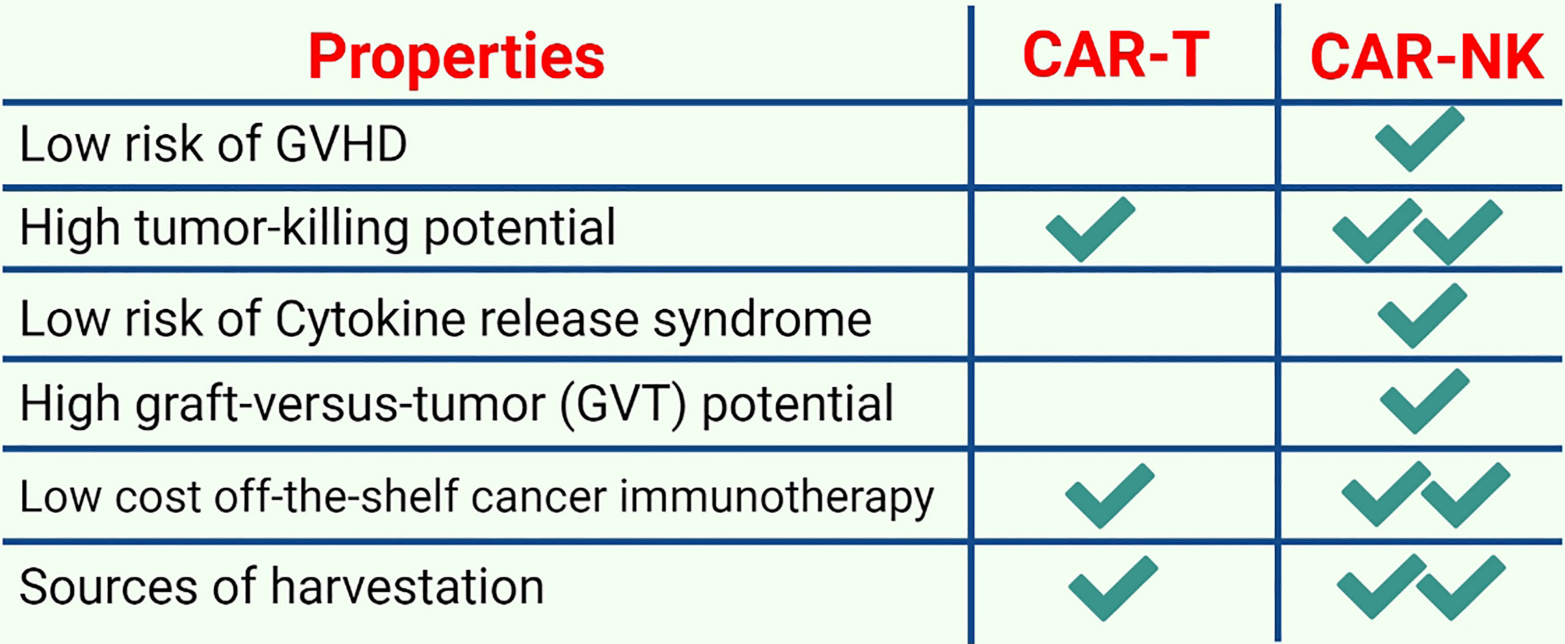
A comparison of CAR-T and CAR-NK immunotherapy: CAR-NK cell therapies are becoming increasingly popular due to several advantageous features such as low safety concerns, low costs, and higher tumor potential.

CAR-NK cell therapy may serve as an alternative to CAR-T therapy in the future because CAR-NK cells possess several unique features detailed below (**Figure 6**).

First, allogeneic haploidentical NK cells are considerably safe for adoptive cell therapy (ACT) because they usually do not mediate and may diminish GVHD (99). Earlier investigations have revealed that NK cells are involved in the induction or aggravation of GVHD. Subsequently, it was observed in both patients and mouse models that NK cells regulate GVHD by suppressing alloreactive T cell responses. NK cells interact with other immune cell subsets during GVHD and suppress GVHD naturally by inhibiting T cell activation *via* their cytotoxic ability unless exogenous hyperactivation causes them to produce proinflammatory cytokines that can sustain T cell-mediated GVHD induction (100). Similarly, CAR-NK cells have considerably fewer safety concerns such as on-target/off-tumor effects, CRS and tumor lysis syndrome (101). For instance, a study that used HSCT to treat AML revealed that NK cells were the main factors in inducing the graft-versus-tumor (GVT) response (35). Moreover, NK cells only secrete a small number of IFN-γ and GM-CSF and do not produce IL-1 and IL-6 that initiate CRS. Second, tumor cells may not be detected by CAR-T cells owing to tumor escape because of a loss of either MHC class I expression or tumor-specific antigens (102). CAR-NK cells lack a self-antigen and can detect MHC class I-negative tumor cells because they retain innate cytotoxic potential against germline-encoded tumor/stress ligands (103). In addition, both HLA-A and HLA-B bind to KIR3D receptors, whereas HLA-C only binds to KIR2D receptors. CD94-NKG2A, which detects HLA-E, LILRB1 and all MHC class I molecules, is another inhibitory receptor that identifies MHC class I molecules expressed by NK cells (104). Normal MHC class I-sufficient cells are ignored by NK cells because their inhibitory receptors can detect MHC class I molecules; however, they are not inhibited after interacting with abnormal MHC class I low cells. Third, it is believed that low levels of MHC class I expression in cancer stem cells (CSCs) and the presence of NKp30, NKp44 and NKG2D (activating receptors) cause cytokine-activated NK cell-mediated death of CSCs (105). CSCs usually use two different mechanisms to escape NK cell detection: shedding the NKG2D ligands MICA and MICB in case of breast CSCs (106) and lacking NKG2D ligands in case of leukemia stem cells (107). Although NK cells can perform serial killing and have a limited life span, cytomegalovirus (CMV)-induced memory-like adaptive NK cells had a prolonged life span and an enhanced cytotoxic potential (108). For instance, after reactivation, NKG2C^+^ NK cells from CMV naïve UCB grafts were found to expand preferentially in recipients, indicating a primary NK cell response after HSCT (109). The effects of donor/recipient CMV serostatus on the expression and activity of NKG2C^+^ NK cells were then evaluated in donor HSCT recipients to identify responses to secondary CMV occurrences. After clinical CMV reactivation, NKG2C^+^ NK cells increased in number. When both the donor and recipient were CMV-seropositive, the cells expanded in the absence of detectable CMV viraemia. CMV-positive recipients who received grafts from CMV-seropositive or -seronegative donors had higher levels of NKG2C^+^ NK cells. These *in vivo*-expanded NKG2C^+^ NK cells had a greater capacity for target cell-induced cytokine release, generated an inhibitory killer Ig-like receptor for self-HLA and acquired CD57 more quickly. Compared with seronegative donors with NKG2C^+^ NK cells, seropositive donors with NKG2C^+^ NK cells responded better to a subsequent CMV infection (110). Fourth, CAR-NK cells can regulate their activating receptors, including NKp30, NKp44, NKp46, NKG2D, KIR-2DS, KIR-3DS, 2B4, CD226, CD94/NKG2C and DNAM-1; therefore, the chances of relapse owing to the loss of CAR-targeting antigens is reduced. Moreover, T lymphocytes only kill their targets through a CAR-specific mechanism, whereas NK cells exhibit spontaneous cytotoxic activity and can kill target cells regardless of the presence of tumor-specific antigens. Tumor cells downregulate antigens to escape immune detection; however, NK cells are still effective against them. Furthermore, cytokines such as IFN-γ, IL-3 and GM-CSF produced by primary human NK cells are different from proinflammatory cytokines released by T cells, which induce CRS. Individual NK cells can survive after interacting with and destroying several target cells, potentially decreasing the number of cells that are adoptively transferred. Fifth, the availability of an off-the-shelf CAR-NK therapy enhances the pace of administration remarkably and first dosing to 1 day by minimizing the lag time from the decision to treat. Sixth, CAR-NK therapy is expected to decrease huge indirect costs because CAR-NK infusions can be administered with outpatient follow-up monitoring and do not require lengthy post-treatment hospitalization because they are safer and have no potential toxicity (111). Although iPSC-derived CAR-T cells can also serve as an off-the-shelf product, they require more rigorous efforts and extra genetic modifications to obtain a universal product that does not require HLA-matching and is devoid of any endogenous TCR. Therefore, iPSC-derived CAR-T cells require post-treatment hospitalization and hence cannot abolish the potential possible side effects associated with CAR-T cell therapy. In addition, NK cells can be harvested from multiple sources including iPSCs, PB, UCB, human embryonic stem cells and NK cell lines (112). Therefore, CAR-NK cells may be a reliable therapeutic candidate to mitigate these limitations and safety concerns.

Recently, a meta-analysis has reported that of the 520 active trials investigating a total of 64 different CARs worldwide, 96.4% of trials are investigating CAR-T cells (96). Therefore, the research on CAR-NK therapy is currently in its beginning stages owing to very few translations of laboratory investigations to clinical settings. Further investigation and clinical trials are required to ensure the safety profile of CAR-NK cells because they have a few side effects and a low incidence of CRS. It is quite delightful to mention that several ongoing clinical trials are investigating the safety and efficacy of CAR-NK cell therapy for both hematological and solid tumors, which are registered on ClinicalTrials.gov (**Table 1**) (113).

**Table 1:** Clinical trials of CAR-NK cell therapies against hematological malignancies and solid tumors.

Serial No.	Tumor Type	Specific Target	Source of NK-cells	Status	Phase	References
1.	Leukemia and lymphoma	CD7	NK-92	Unknown	I/II	NCT02742727
2.	Leukemia and lymphoma	CD19	NK-92	Recruiting	I/II	NCT02892695
3.	Leukemia and lymphoma	CD19	Umbilical cord blood	Recruiting	I/II	NCT03056339
4.	Leukemia and lymphoma	CD19	Umbilical cord blood	Withdrawn	I/II	NCT03579927
5.	Acute myeloid leukemia	CD33	NK-92	Unknown	I/II	NCT02944162
6.	Acute myeloid leukemia	CD19	Expanded donor NK cells	Completed	I	NCT00995137
7.	Acute myeloid leukemia	CD19	Haploidentical donor NK cells	Suspended	II	NCT01974479
8.	Relapsed & Refractory Acute myeloid leukemias	CD33	NK-92	Unknown	I/II	NCT02944162
9.	Relapsed & Refractory B Cell Lymphoma	CD19	Unknown	Not Yet Recruiting	Early phase I	NCT03690310
10.	Relapsed & Refractory B Cell Lymphoma	CD22	Unknown	Not Yet Recruiting	Early phase I	NCT03692767
11.	Relapsed & Refractory B Cell Lymphoma	CD19/CD22	Unknown	Not Yet Recruiting	Early phase I	NCT03824964
12.	Multiple Myeloma	BCMA	NK 92	Recruiting	I/II	NCT03940833
13.	Pancreatic Cancer	ROBO1	Unknown	Recruiting	I/II	NCT03941457
14.	Epithelial ovarian cancer	Mesothelin	Unknown	Not Yet Recruiting	Early phase I	NCT03692637
15.	Castration-resistant Prostate Cancer	PSMA	Unknown	Not Yet Recruiting	Early phase I	NCT03692663
16.	Non-small cell lung cancer	Unknown	CCCR-NK-92	Recruiting	I	NCT03656705
17.	Glioblastoma	HER2	NK-92	Recruiting	I	NCT03383978
18.	Solid tumors	MUC1	Unknown	Unknown	I/II	NCT02839954
19.	Solid tumors	NKG2D ligands	Autologous or allogeneic NK cells	Recruiting	I	NCT03415100
20.	Solid Tumors	ROBO1	Unknown	Recruiting	I/II	NCT03940820

## Preclinical Applications of CAR-NK Therapy

Allogeneic stem cell transplantation of NK cells has shown significant success for the treatment of AML in preclinical trials (114). However, a few limitations at the clinical level need to be overcome, including the moderate activity of NK cells and tumor escape from immune surveillance (115). Therefore, CAR was proposed for reprogramming NK cells to enhance their efficacy and cytotoxicity against tumors. Currently, a large number of ongoing studies use CAR-modified NK cells and NK-92 cell lines against various types of tumors (116).

### Preclinical Successes in Hematological Tumors

Recently, Romanski et al. (117) successfully improved the sensitivity and cytotoxic potential of NK cells against B-lineage malignant cells by constructing NK92-CD19-CD3ζ cells (117). Shimasaki et al. (118) successfully expressed CD19-41BB-ζ on NK cells employing electroporation technology for transfecting its mRNA (118). Subsequently, CAR-NK cell therapy was found very effective, *via* optical *in vivo* imaging, in reducing the growth of leukemia xenografts. Moreover, to improve the cytotoxicity of NK cells against rituximab-resistant Burkitt lymphoma, Chu et al. (119) successfully transfected CD20-BB-ζ mRNA in NK cells through nuclear transfection and co-cultivated them with K562-mb-IL15-41BBL for activating CAR-NK cells (119). Furthermore, CD138-CAR-NK-92 has also been reported to be very effective in improving the survival rate of mice with MM (120). Notably, the effective elimination of EBNA3C-expressing Epstein–Barr virus-positive T cells by CAR-NK92 cells indicates a remarkable cytotoxic potential of CAR-expressing NK cells (121). Similarly, Liu et al. (122) recently used CB-derived HLA-mismatched anti-CD19 CAR-NK cells to treat relapsed or refractory CD19-positive cancers. They found that CAR-NK cell administration was well tolerated and did not lead to CRS, neurotoxicity, GVHD and elevated inflammatory cytokines such as IL-6. Of the 11 patients, 8 exhibited a response to the therapy, 7 had complete remission, whereas 1 had remission of the Richter’s transformation component but had persistent chronic lymphocytic leukemia (122). Recent preclinical studies using CAR-NK cells against various hematological cancers are summarized in **Table 2**.

**Table 2:** Recent preclinical studies employing CAR-NK cells against various hematological cancers.

Serial No.	Tumor Type	Specific Target	Source of NK-cells	CAR composition	Clinical Outcomes	References
**1.**	T cell malignancies	CD5	NK-92	CD28+4-1BB+CD3ζ	Inhibition and control of disease progression	(123)
**2.**	B-cell malignancies	CD19	PB-NK	CD28+4-1BB+CD3ζ	Complete elimination of leukemia after 48 h	(124)
**3.**	B-cell malignancies	CD19	PB-NK	4-1BB+CD3ζ	Augmented cytotoxicity of NK cells	(118)
**4.**	B-ALL	CD19	PB-NK	CD28+CD3ζ	Complete and durable molecular remissions of pre-B-ALL	(125)
**5.**	B-cell malignancies	CD19	NK-92	CD3ζ	Successful inhibition of disease progression	(126)
**6.**	B-cell malignancies	CD19	NK-92	CD3ζ	Overcame NK resistance and markedly enhanced NK-cell-mediated killing	(98)
**7.**	B-cell malignancies	CD19	PB-NK	CD28+CD3ζ+IL15	Superior cytotoxicity with up to 90% specific killing activity	(127)
**8.**	B-cell malignancies	CD19	NK-92	41BB-CD3ζ,	Specific and efficient lysis of leukemia cell lines and lymphoblasts	(117)
**9.**	B-ALL	FLT3	NK-92	CD28+CD3ζ+iCasp9	Remarkable inhibition of disease progression and high antileukemic activity	(128)
**10.**	B-cell malignancies	CD19	UCB-NK	4-1BB+CD3ζ+iCasp9+IL15	Efficient killing of CD19-expressing cell lines and primary leukemia cells with marked prolongation of survival	(129)
**11.**	B-cell malignancies	CD20	PB-NK	4-1BB+CD3ζ	Significantly enhanced cytotoxicity and IFNγ production, extended survival time and reduced tumor size	(130)
**12.**	Burkitt lymphoma	CD20	PB-NK	4-1BB+CD3ζ+IL15	Significant anti-tumor effects and enhanced *in vitro* cytotoxicity	(119)
**13.**	MM	CD-38	NK-92	CD28-41BB-CD3ζ	Specific lysis of CD38-expressing tumor cell lines and effective depletion of MM	(131)
**14.**	MM	CD138	NK-92MI	CD3ζ	Remarkable cytotoxicity against human MM cell lines and elevated secretion of granzyme B, interferon-γ and CD107a proportion	(120)
**15.**	MM	CS-1	NK-92	CD28+CD3ζ	Enhanced MM cytolysis and IFN-γ production, efficient suppression of human IM9 MM cells and significant survival of mice	(132)
**16.**	Peripheral T cell lymphoma	CD-4	NK-92	CD28-41BBCD3ζ	Specific elimination of T-cell leukemia, lymphoma cell lines, and patient samples *ex vivo*	(133)
**17.**	T cell malignancies	CD-5	NK-92	2B4-CD3ζ	Specific cytotoxicity against CD5^+^ malignant cells and prolonged survival of T-ALL xenograft mice	(134)
**18.**	T-ALL	CD-7	NK-92	CD28-41BBCD3ζ	Potent anti-tumor activity, elevated Granzyme B and IFNγ secretion, and significant inhibition of disease progression	(135)
**19.**	EBV+ T cell	EBNA3C	NK-92	4-1BB+CD3ζ	Exquisite specificity, potent cytotoxicity, and induction of ADCC toward the targeted T-cell epitope (TCE)	(121)
**20.**	Non-Hodgkin’s lymphoma or chronic lymphocytic leukemia	CD-19	UCB-NK	CD28+CD3ζ+ IL15+iCasp9	complete remission in 7/11 (4 with lymphoma and 3 with CLL)	(122)

### Preclinical Success in Solid Tumors

The success of CS1-CD28/CD3ζ-NK92 cells in restricting the growth of MM, enhancing IFN-γ production and improving the survival rate (132) has encouraged oncologists to design CAR-NK immunotherapies against solid tumors. Consequently, CAR-NK-92 cells expressing EGFR-CD28-CD3ζ exhibited remarkable cytotoxicity and killing potential in glioblastoma (GBM) cells (136). Similarly, GD2-specific NK-92 cells were found to exert cytolysis and effectively eliminate neuroblastoma (137). NKG2D-DAP10-CD3ζ-expressing NK-cells were found to exhibit a remarkably strong antitumor potential in several different cancers including breast cancer, hepatocellular carcinoma (HCC), osteosarcoma and pancreatic cancer (97). Prostate stem cell antigen (PSCA)-DAP12 CAR-expressing PB-NK and YTS-NK cells were found highly beneficial against PSCA^+^ tumors (138). A significantly high expression of human epidermal growth factor receptor 2 (HER2) in GBM (139), renal cell (140) and breast cancer (141) makes it an ideal candidate to develop immunotherapy using HER2-CAR-modified NK cells. CAR-NK92 cells designed to target epithelial cell adhesion molecules (EpCAMs) were highly efficient in killing breast cancer cells (142). The growth of ovarian cancer xenografts was remarkably reduced in mice after treatment with CAR-iPSC-NK cells (143). It is noteworthy that following a standard protocol, iPSC-derived NK cells can be synthesized on a larger scale (144), leading to feasible administration of multiple doses to treat refractory solid tumors more efficiently. Recent preclinical studies using CAR-NK cells against various solid tumors are summarized in **Table 3**.

**Table 3:** Recent preclinical studies employing CAR-NK cells against various solid tumors.

Serial No.	Tumor Type	Specific Target	Source of NK-cells	CAR composition	Clinical Outcomes	References
1.	Osteosarcoma/Prostate/HCC/Breast cancer	NKG2D	PB-NK	NKG2D+DAP10+CD3ζ	Enhanced cytotoxicity and secretion of IFN-γ, GM-CSF, IL-13, MIP-1α, MIP-1β, CCL5, and TNF-α, and cytotoxic granules which persisted after 48h	(97)
2.	Multiple solid tumors	NKG2D	NK-92	DAP10+CD3ζ	Enhanced anti-tumor cytotoxicity both *in vitro* and *in vivo*	(145)
3.	Ovarian cancer	NKG2D	PBMCs	CD8α+ CD3ζ	Augmented tumor infiltration and significant antitumor responses	(146)
4.	Bladder carcinoma	PSCA	YTS/PB-NK	DAP12	Improved cytotoxicity, delayed tumor growth and complete tumor eradication	(138)
5.	Breast carcinoma	EpCAM	NK-92/NKL	CD28+CD3ζ+IL-15	Predominantly intracellular expression of the cytokine, and STAT5 activation, high and selective cell-killing activity	(142)
6.	Colorectal cancer	EGFRvIII	NK-92	CD8α+ CD28+ CD3ζ	Development of a sensitive *in vitro* platform to evaluate CAR efficacy	(147)
7.	Breast cancer	EGFR	PB-NK/NK-92	CD28+CD3ζ+oHSV	Enhanced cytotoxicity and IFN-γ production, efficient killing and significantly longer survival	(148)
8.	Renal cell carcinoma	EGFR	NK-92	CD28+4-1BB+CD3ζ	Potent antitumor activity and long-lasting immunological memory	(77)
9.	GBM	EGFRvIII	YTS	DAP12	Specific cytotoxicity, significantly delayed tumor growth, increased survival, and complete tumor remission	(149)
10.	GBM	EGFR/EGFRvIII	NK-92/NKL	CD28+CD3ζ	Enhanced cytolytic capability, IFN-γ production, efficient suppression of tumor, and significantly prolonged the survival	(136)
11.	GBM	EGFR/EGFRvIII	NK-92	CD28+CD3ζ	High and specific cytotoxicity and tumor lysis, and marked extension of survival	(150)
12.	Melanoma	GPA7	NK-92	HLA-A2TM+CD3ζ	Enhanced tumor killing and suppression of the growth of human melanoma	(151)
13.	GBM/breast cancer	HER2	NK-92	CD8α+CD3ζ	Specific and efficient tumor lysis	(152)
14.	GBM/breast cancer	HER2	NK-92	CD28+CD3ζ	Potent *in vivo* antitumor activity, marked extension of survival	(139)
15.	Breast cancer/renal cell carcinoma	HER2	NK-92	CD28+4-1BB+CD3ζ	Efficient *in vitro* lysis, serial target cell killing, and reduction of metastasis	(140)
16.	Neuroblastoma	GD2	NK-92	CD3ζ	Remarkable cell killing activity	(137)
17.	Ewing sarcomas	GD2	PB-NK	CD28+4-1BB+CD3ζ	Enhanced *in vitro* responses and overcame resistance to NK cell lysis	(116)
18.	hepatocellular cancer	Glypican-3 (GPC3)	NK-92	CD28+41BB+CD3ζ CD3ζ CD28+CD3ζ DNAM1+CD3ζ DNAM1+2B4+CD3ζ	More quick expansion, more persistence, and higher cytotoxicity	(153)
19.	Ovarian cancer	Glypican-3 (GPC3)	iPSC	CD8α+CD28+CD137+CD3ζ,	Enhanced cytotoxicity, IFN-γ production, and prolonged the survival	(154)
20.	Ovarian cancer	Mesothelin	iPSC	2B4+CD3z	Superior anti-tumor potential, significant inhibition of tumor growth and prolonged survival	(143)
21.	Ovarian cancer	Mesothelin	NK-92	CD28+41BB+CD3ζ	Specific *in vitro* killing, enhanced cytokine production, efficient tumor elimination, and prolonged survival	(11)
22.	Prostate Cancer	Prostate Stem Cell Ag (PSCA)	YST cell line, primary NK	DAP12	Improved cytotoxicity, delayed tumor growth and complete tumor eradication	(138)
23.	Colorectal Cancer	Carcinoembryonic antigen (CEA)	NK-92	CD3ζ	Improved recognition and lysis of the tumor cell lines	(155)
24.	ovarian cancer	CD133	NK-92	CD28-41BBCD3ζ	Enhanced cytotoxicity and IFN-γ production	(156)
25.	Liver cancer	c-MET	Peripheral blood	41BB+DAP12	Improved specific cytotoxic potential	(157)
26.	PD-L1^+^Solid tumors	PD-L1	NK-92	41BB	Improved antitumor potential and significant inhibition of tumor growth	(158)
27.	Triple-negative breast Cancer	Tissue Factor (TF)	NK-92	CD28+41BB+CD3ζ	Superior tumor killing	(159)

## Clinical Applications of CAR-NK Therapy

A large number of studies have been conducted on CAR-T cells; however, only a few clinical trials on CAR-NK cells (**Table 1**) have been registered on ClinicalTrials.gov. In addition to CD19 (NCT02742727), CD7 (NCT02742727) and CD33 (NCT02944162) are also the prime targets for CAR-NK cell therapy in clinical studies on lymphoma and leukemia. Furthermore, HER2-targeted GBM (NCT03383978) and costimulating conversion receptors are under investigation clinically to treat non-small-cell lung carcinoma (NSCLC) (NCT03656705). CAR-NK cell therapy against multiple refractory solid tumors targeting mucin 1 (MUC1), including pancreatic tumors, HCC, NSCLC and triple-negative invasive breast tumors, is also under investigation in clinical trials (NCT02839954). The details of ongoing clinical trials of CAR-NK cell therapies against hematological malignancies and solid tumors are summarized in **Table 1**.

## Current Limitations of CAR-NK Cell Therapy

### Low Persistence

The lack of *in vivo* durability of infused cells in the absence of cytokine support is one of the key drawbacks of adoptive NK cell treatment. Although it may be safe, it may limit the efficacy of NK cell immunotherapy. Exogenous cytokines have been demonstrated to increase the proliferation and durability of adoptively infused NK cells; however, they can also cause undesired side effects (160), including the growth of inhibitory immune subsets such as Tregs (161). Multiple studies have reported promising results by engineering NK cells with transgenes encoding for cytokines that are either expressed on the membrane or released constitutively. In one such study, tumor-harboring mice with NK-92 cells or primary NK cells transduced with retroviral vectors producing IL-2 or IL-15 had increased proliferation and persistence (162). It has also been demonstrated that integrating IL-15 transgene into a CAR construct improves NK cell proliferation, *in vivo* persistence and antitumor activity in patients with high-risk lymphoid malignancies, without increasing systemic levels of IL-15 or causing toxicity (122, 129). Other armored CAR-NK cells with cytokine transgenes are under development; however, there are no published reports yet. Another way to increase NK cell persistence is by inducing a memory-like phenotype, such as by preactivating them with a cytokine cocktail (IL-12, IL-15 and IL-18) for a brief period to induce differentiation into cytokine-induced memory-like NK cells (64, 163). Memory-like NK cells were recently modified to express a CAR directed against CD19 and showed improved responses *in vitro* and *in vivo* against NK-resistant B-cell lymphoma (164).

### Transport to the Required Tumor Site

Rapid homing to tumor beds is essential for adoptive cellular treatment efficacy and is governed by complicated interactions between chemokines released by NK cells and tumor cells (165). The efficiency of NK cell homing to tumor sites has been controversial, thus prompting efforts to improve it (166). The chemokine receptor CCR7 was transferred from K562 feeder cells to NK cells through trogocytosis, which resulted in improved homing of NK cells to the lymph nodes (167). In a xenograft-harboring mouse model of CXCL10-transfected melanoma, overexpression of CXCR3 on NK cells after *ex vivo* growth with irradiated EBV-LCL feeder cells and IL-2 resulted in better trafficking and antitumor activity (168). Several researchers have since investigated various engineering methods to improve NK cell homing. For example, NK cells were electroporated with mRNA coding for the chemokine receptor CCR7 to increase movement toward the lymph nodes that express the chemokine CCL19 (169). NK cells transduced with a viral vector encoding CXCR2 demonstrated better motility to renal cell carcinoma tumors expressing cognate ligands such CXCL1, CXCL2, CXCL5, CXCL6 and CXCL8 (170). The NK cell-recruiting protein-conjugated antibody (NRP-body) with a cleavable CXCL16 molecule was used in another study to increase NK cell trafficking and penetration into pancreatic tumors (171). Furin, an endoprotease expressed on the surface of pancreatic cancer cells, cleaves CXCL16, thus promoting NK cell infiltration *via* the ERK signaling cascade. In a mouse model of pancreatic cancer, this method was demonstrated to improve NK cell-mediated tumor suppression (171). CAR-NK cells have also been modified to improve their ability to travel to the tumor sites. Müller et al demonstrated that anti-EGFRvIII CAR-NK cells modified to produce CXCR4 conferred selective chemotaxis to CXCL12/SDF1-secreting glioblastoma cells in a mouse model of glioblastoma, leading to better tumor regression and survival (149). Furthermore, in mice with established peritoneal ovarian cancer xenografts, NKG2D CAR-NK cells modified to express CXCR1 significantly increased antitumor responses (146). To improve the success of NK cell immunotherapy in patients with solid tumors, several novel techniques to promote NK cell trafficking to tumor sites have been investigated in mice models; however, the efficacy of these approaches should be validated in clinical trials.

### Immunosuppressive Tumor Microenvironment

TME, which includes immunosuppressive soluble chemicals, immunosuppressive cells and an unfavorable environment for optimal immune cell function, is a major barrier to successful CAR-NK cell therapy. TGF-β; adenosine; indoleamine 2,3-dioxygenase (IDO) and prostaglandin E2 (PGE2) are immunosuppressive cytokines and metabolites found in TME that can impair NK cell activity (23). Treg cells; regulatory B cells; myeloid-derived suppressor cells (MDSCs); tumor-associated macrophages (TAM); platelets; fibroblasts and several unfavorable metabolic factors such as hypoxia, acidity and nutrient deprivation induce immunosuppression in the malignant milieu (172, 173). Therefore, researchers are working towards developing CAR-NK cells that can prevent some of these immunosuppressive effects. Engineering NK cells to make them resistant to TGF-β has shown to be a promising strategy. The TGF-β receptor 2 (TGFβR2) gene was deleted using CRISPR/Cas9 technology in primary human NK cells, rendering them immune to this immunosuppressive growth factor without losing their efficacy against AML (174). Similarly, NK cells that were genetically engineered to express a dominant-negative TGF-β receptor, a high-affinity non-signal transducing receptor generated from TGFβR2, were able to counteract the suppressive effects of TGF-β on NK cells and restore their cytotoxicity (175). An anti-miRNA against miR-27a-5p, an miRNA that is elevated by TGF-β in NK cells, improved NK cell effector function both *in vitro* and *in vivo* (176). Furthermore, adenosine, an important immunosuppressive metabolite generated from ATP by the ectonucleotidases CD73 and CD39 in response to hypoxia and stress (177), has been targeted by blocking the high-affinity A2A adenosine receptor on NK cells, resulting in more potent antitumor activity in mouse models of breast cancer, melanoma and fibrosarcoma (178, 179). Another important method by which TME causes NK cell failure is checkpoint molecule interaction (30). To overcome this problem, genome editing is employed to eliminate checkpoint components from NK cells to improve their function. In tumor-harboring mice, TIGIT deletion was demonstrated to protect against NK cell depletion and enhance prognosis (180). Some researchers have investigated NKG2A and found that NKG2A^null^ NK cells exhibit increased cytotoxicity against HLA-E-expressing malignancies (181). Recent studies have suggested that combining CAR engineering with checkpoint deletion is effective in enhancing NK cell antitumor activity (by targeting CIS, a negative regulator of cytokine signaling) (182–184). The classic T cell checkpoints PD-1 and CTLA4 are two other inhibitory molecules that are under investigation in NK cells (185, 186). The use of checkpoint blockade to improve NK cell effector activity has recently been discussed in detail (187). Literature review reveals that creative engineering techniques and genome editing technologies may overcome the biological limitations of NK cells and hurdles caused by TME. Adoptive therapy with NK cells is likely to transform from a safe treatment with only moderate efficacy to a serious contender as a first-line treatment in cancer immunotherapy if strategies are developed to improve NK cell persistence, trafficking to tumor sites, and effector function in a hostile and malignant milieu.

### Low Lentivirus Transduction Efficiency

Lentivirus-based transduction system is one of the most common approaches for gene modification and delivery in cells. However, owing to native characteristics, NK cells are resistant to lentivirus, which makes lentivirus-based transduction a challenge. To improve viral transduction, various chemicals have been used. For instance, the electrical charge on cell membranes can be removed using protamine sulfate or polymers (dextran or polybrene) (188). Similarly, in HSCs and progenitor cells, cyclosporine A (189) and rapamycin (190) may help in removing different lentiviral restriction barriers. Interestingly, inhibition of intracellular antiviral defense mechanisms was reported to enhance the efficiency of lentiviral transduction in human NK cells (191). Furthermore, vectofusin-1 (192), prostaglandin E2 (188) and dextran (190) were found to promote transduction rates in human HSCs, T lymphocytes and primary NK cells, respectively (193). Furthermore, rosuvastatin has been discovered to increase the effectiveness of VSV-G lentiviral transduction of NK cells by upregulating LDLR levels (194). In addition, Colamartino ABL et al. (195) have revealed an effective and a resilient approach for NK cell transduction using baboon envelope pseudotyped lentivectors (BaEV-LVs) (195). They observed a transduction rate of 23.0 ± 6.6% and 83.4 ± 10.1% (mean ± SD) in freshly isolated human NK cells and those from the NK cell activation and expansion system (NKAES), respectively. Furthermore, CAR-CD22 transduced with BaEV-LVs exhibited robust CAR expression on 38.3 ± 23.8% (mean ± SD) of NKAES cells and particularly destroyed the NK-resistant pre-B-ALL-RS4;11 cell line. A larger vector encoding a dual CD19/CD22-CAR and a low viral titre were used to accomplish successful transduction and re-expansion of dual-CAR-expressing NKAES for efficient killing of both CD19^KO^- and CD22^KO^-RS4;11 cells (195). In addition, Bari R et al. (196) found that lentiviral vectors pseudotyped with a modified baboon envelope glycoprotein had a 20-fold or higher transduction rate than that of a VSV-G pseudotyped lentiviral vector (196). Moreover, using CD19-CAR, they achieved efficient and specific killing of CD19-expressing cell lines.

## Future Strategies to Overcome These Limitations/Future Perspectives

Although CAR-NK cells are optional, potentially competitive cancer immunotherapeutic candidates along with CAR-T cells, numerous obstacles including heterogeneity, low persistence, trafficking to the tumor site, hostile TME and loss of tumor antigens remain to be overcome.

The most critical step in designing CARs is to identify highly and uniformly expressed target tumor antigens. Most tumor-associated antigens (TAAs) are also expressed by several healthy cells; therefore, achieving ‘on-target, off-tumor’ effects is inevitable (197). Moreover, huge differences may be observed in the expression of these TAAs among single-cell clones from the same tumor owing to two major strategies to evade immune surveillance—clonal evolution and decreased TAA expression. To overcome this problem, bispecific CARs were designed to target two different antigens simultaneously in prostate cancer, and very encouraging results were obtained (198).

Similarly, to overcome difficulties in the accessibility or trafficking of CARs in solid tumors, several approaches are used, including local administration, intraperitoneal administration and focused ultrasound-guided delivery. For instance, pleural injection was found very effective in an orthotopic model mimicking human pleural malignancies with even longer functional persistence than that obtained with intravenous injection (199). Regional administration of CAR-based immune cells may also help in reducing the therapeutic dose. Moreover, anti-HER2 CAR-NK-92 cells have been administered, using focused ultrasound, into the brain of mice with metastatic breast cancer (200). To minimize significant tissue damage caused by CAR-NK cells, ultrasound bursts along with intravenous injection of microbubbles were passed through the intact skull, allowing the temporary passage of NK-92 cells through the blood–brain barrier.

Tumors are equipped with several immunosuppressive factors such as TGF-β, IL-10, PD-1 or arginase. There are several ways to reduce the inhibitory effects of TGF-β. For instance, the combination of TGF-β kinase inhibitors and NK cells has been found to retain the cytotoxicity and expression of NKG2D and CD16 (201). Similarly, the use of either fresolimumab (TGF-β neutralizing antibody) or galunisertib (TGF-βRI inhibitor) has shown very encouraging results owing to their safety and tolerability in solid tumors (29). Moreover, the use of hybrid CARs equipped with an extracellular TGF-β receptor domain has been found quite successful in improving the antitumor potential of NK-92 cells (202). In addition, the cytotoxic activity of NK cells has been enhanced successfully by knocking down SMAD3 (downstream mediator of TGF-β) in solid tumors (145). Similarly, the expression of a dominant-negative TGF-β receptor has been found quite effective in retaining the ability of UCB-NK cells to produce IFN-γ and kill glioblastoma cells. Entinostat (a histone deacetylase inhibitor) has been reported to enhance MICA expression on cancer cells and NKG2D in primary NK cells, resulting in improved tumor cell recognition, and induce NK cytotoxicity despite the hypoxic conditions of tumors (203).

TME is further characterized by the scarcity of nutrients and significant hypoxia that lead to acidosis, eventually suppressing the immune responses (204). Hypoxia helps in tumor development by disturbing metabolism and enhancing the expression of several tumor growth factors and angiogenesis. Moreover, hypoxia promotes tumor growth and metastasis by decreasing the expression of several NK cell-activating receptors including NKG2D, NKp30, NKp44 and NKp46 (205). In addition, CD73 has been found to induce arginase (an immunosuppressive metabolite) in the hypoxic state to block the NK cell activity. Researchers have demonstrated an improved antitumor activity by enhancing the homing of NKG2D-CAR-NK cells to tumor sites in lung cancer (206).

Several immune checkpoints regulate and inhibit NK cell activity. These immune checkpoints act as ‘a natural brake’ to prevent autoimmune diseases or immuno-pathological conditions caused by overactivation. Cancer cells can evade immune surveillance by expressing several checkpoint proteins that inhibit or block immune cell activation. Genetic deletion or blockage of these checkpoints can help CAR-NK cells to remain hyperactive and get rid of cancer and metastases more quickly. For instance, TIGIT has been found to prevent the cytotoxicity of NK cells by opposing CD226 (207). Moreover, decreased proliferation and effector potential was observed in PD-1^+^ NK cells, whereas an improved effector activity was observed in PD-L1^+^ NK cells (208). Subsequently, the reactivation of exhausted immune cells and long-lasting clinical outcomes have successfully been achieved by inhibiting PD-1 or PD-L1 *via* checkpoint blocking agents (209). Furthermore, persistent therapeutic benefits have been observed with a combination of CAR and checkpoint proteins (PD-1, CTLA-4, LAG3 and TIGIT) blockers (198). NK-92 cells expressing CD16, IL-2 and PD-L1-specific CARs have been found to destroy several human cancer cells, such as breast, lung and gastric cancer cells, by secreting a large number of perforin and granzymes (210). Interestingly, the use of antibodies as checkpoint inhibitors is under development in several clinical trials. For instance, two mAbs, namely, lirilumab (IPH2101 or 1-7F9) and IPH4102, have recently been developed to specifically target KIRs and KIR3DL2, respectively. Lirilumab has been engineered to target the common epitope shared by KIR2D that renders alloreactivity to NK cells to kill cancer cells by disrupting the inhibitory KIR-L/HLA interactions. The use of this antibody for NK stimulation in combination with lenalidomide has been found quite safe, tolerable and clinically effective against MM in a phase I trial (211). In addition, IPH4102 has been used for the treatment of cutaneous T cell lymphomas because these malignancies have a higher expression of KIR3Dl2 (212). This treatment has been found well tolerated and clinically effective in phase I trials, and the results are very encouraging and will prompt further large-scale clinical investigations (213).

Another important strategy to enhance the activity of CAR-NK cells that has not received the required attention is the modulation of tumor metabolism. Under hypoxic conditions, adenosine is produced by metabolizing ATP *via* CD39 and CD73, which are involved in immune evasion, blocking NK cell transportation to tumor sites, and preventing NK cell maturation (206). Moreover, the use of anti-CD39 and anti-CD73 antibodies to inhibit adenosine has been found quite successful in enhancing the effects of targeted therapy for ovarian cancer (214). CD73 may be an important target to treat several solid tumors such as glioblastoma, prostate cancer and lung cancer because it is highly expressed in these tumors. NKG2D-engineered CAR-NK cells have shown promising effects in treating lung cancer after anti-CD73 antibody-based inhibition (206).

To overcome the antigen loss after CAR therapy, more than one antigen can be targeted simultaneously. This can be achieved in many ways; for instance, different CARs targeting different antigens can be injected simultaneously (215); another approach may involve the use of vectors for two CARs that can be combined and used during the cell production step to obtain a mixture of cells equipped with single CARs and for both CARs as well. However, high costs involved in making multiple vectors and heterogeneity of CARs resulting in poor clinical analysis remain the major drawbacks of this strategy. Another important approach is to design a CAR that can recognize multiple antigens. This goal can be achieved *via* ‘tandem CAR’, where two binders are attached to a single molecule to improve the efficiency of immune synapse. In addition, the ribosomal skip sequences of internal ribosomal entry sites can help in generating multiple CARs on the same immune cell using a single vector, which is called ‘bicistronic CAR’. Bielamowicz et al. recently targeted three different antigens on glioblastoma using a trivalent vector that encoded three independent CARs (216). It is quite promising that, recently, the number of trials using CARs that target multiple antigens simultaneously has increased. We anticipate that more trials will investigate CARs capable of simultaneously targeting two or more antigens in the future.

Another important strategy is to increase CAR-NK cell activation. A prime target for NK cell activation is CD16 that can induce the killing effect upon engagement. Identification of more such proteins/receptors for CAR-NK cells may enhance the efficacy of CAR-NK therapies. Other significant approaches to increase the safety profile of CAR-based NK cell therapies may involve the modification of CAR constructs by incorporating suicide genes (217) or developing bispecific CAR molecules to better target the tumor-specific antigens (150). Interestingly, CAR-NK cells can equally target tumors in CAR-dependent and CAR-independent manners; therefore, this property of NK cells should be conceivably used to exert an enhanced tumor-killing effect and develop non-signaling CARs. These non-signaling CARs lack direct killing signals but can enhance the legitimate killing technique of NK cells by promoting dwelling and adherence of these cells to targets (218). Another interesting strategy is to design CARs that can modulate or reprogramme the local TME via either immunosuppression or immunoactivation. One such CAR-based NK cell has already been developed and named either ‘armored’ CAR-NK cells or ‘NK cell pharmacies’. These very special CAR-NK cells express several exogenous genes that can modulate the local TME to prevent any harmful effects (219). In line with it, a coalescence of CAR-based NK cell therapy and several alternative therapies might be a very effective option for efficiently eradicating the tumors. For instance, several chemicals can be used for immunosuppression before CAR-NK cell infusion to prevent or lagging the rejection of NK-cell by the host’s defense system. Similarly, CAR-NK cell therapies could be made more effective in combination with radiotherapy. It has been previously proved that radiotherapy, especially stereotactic body radiotherapy (SBRT), helps in boosting the efficacy of immunotherapy (220). Radiations result in DNA damage that induces NKG2D expression on cancer cells and paves the path for NK cell activation and consequently killing effect. Hence, a combination of CAR-NK cell therapy and radiotherapy could be a sound option in lieu of targeting excrescences.

A large number of clinical investigations have been conducted using adoptive cell transfer of autologous NK cells for the eradication of several tumor types such as lymphoma, breast cancer, colon cancer, and lung cancer (221). Nevertheless, the results were not satisfactory with poor antitumor activities due to the interplay of inhibitory receptors that are found on NK cells and self MHC class I that are found on cancer cells. This self-recognition prevented the stimulation of NK cells (222). For instance, mature NK cells bear a short lifespan at the point of malignancy, thus no long-term adverse effects were seen. Nonetheless, other kinds of NK cells, for instance, that are generated through cord blood or HSCs, have a longer lifespan and may cause long-term damage (223). To overcome this problem, researchers are investigating a new way for integrating caspase-controlled suicide vectors keen on CAR-NK cells, which might swiftly eliminate those cells that are transduced. In this regard, a contemporary investigation has shown that the addition of the matching small molecule dimerizer to the persuadable caspase 9 (iCAS-9) suicide schemes in CD19-CAR+IL15 NK cells caused apoptosis in 4 hours (129). HLA-mismatch donor haematopoietic transplantation in AML patients might prevent relapse and graft rejection without GVHD due to the donor-recipient NK cell alloreactivity that comes from KIR-ligand incompatibility (35). As several clinical investigations have highlighted the significance of KIR-HLA interactions in HSCT (224), KIR genotyping in the near future can serve as an important factor in donor selection. The cost of KIR genotyping is quite competitive and easy to perform; hence, can be done for donor screening together with HLA genotyping. Indeed, several trials that employ KIR genotyping for donor selection are in progress. Therefore, this approach can also opt for future target-oriented CAR-based immunotherapies.

Furthermore, KIR2DSs and KIR3DSs use ITAM (DAP-12) for the phosphorylation of tyrosine residue and recruitment of ZAP-70 or Syk that enhances NK cell activation and NK cell recognition of the tumor cells (225). Therefore, this potential of KIRs could be exploited for improved and highly efficient CAR-NK-based immunotherapies.

Adoptive immunotherapies are usually accompanied by certain side effects. One approach to minimize the risk associated with the adoptive immunotherapies is to endow the immune cells to target tumor-specific neoantigens. As the antigen screening technologies are progressing, more ways to identify the tumor neoantigens are being employed including inventory-shared neoantigen peptide library, whole-exome sequencing in combination with mass spectrometry, and neoantigen detection *via* trogocytosis. Hence, the future CAR-NK immunotherapies can be improved by employing this approach, to better treat the tumors resistant to conventional anti-cancer therapies.

Collectively, progress and advancement in the NK cell immunobiology field have led down the base of better and novel immune therapies. Excellent antitumor bloodlines of the NK cells have made them the center of focus of cell-based immunotherapies. Especially, the HLA phenotype independent NK cell recognition can be exploited to develop NK cell banks instead of modified CAR-NK cells. Subsequent CAR-NK cells are promising as novel anti-cancer therapies that could serve as “off-the-shelf” products. Advancements in the field of gene manipulation, antigen-screening technologies, and KIR-typing have allowed the development of novel, more powerful, and target-oriented CAR-NK cells with strong anti-tumor potential. Similarly, the use of bispecific CARs and Tandem CARs, genetic deletion/blocking checkpoint inhibition, and modulating tumor microenvironment are few other strategies that can better treat several tumor types. With an enhanced safety profile and promising success of CAR-NK immunotherapies in preclinical studies and clinical investigations, together with impressive efforts to overcome the existing challenges, we will witness progress and improvements in cancer treatment in the near future.

## Author Contributions

MK collected the data, draw figures, and wrote the manuscript. HS proposed the idea, modified, supervised, and approved the final version of the manuscript. All authors contributed to the article and approved the submitted version.

## Funding

This study was funded in part through the Startup Foundation for Advanced Talents and Science and Technology Innovation Foundation at Yangzhou University (HS).

## Conflict of Interest

The authors declare that the research was conducted in the absence of any commercial or financial relationships that could be construed as a potential conflict of interest.

## Publisher’s Note

All claims expressed in this article are solely those of the authors and do not necessarily represent those of their affiliated organizations, or those of the publisher, the editors and the reviewers. Any product that may be evaluated in this article, or claim that may be made by its manufacturer, is not guaranteed or endorsed by the publisher.
